# Account of Diphtheritis, as It Occurred on the Watershed between the Tallahatchie and Mississippi Rivers

**Published:** 1860-08

**Authors:** Lea Z. Williamson

**Affiliations:** Sardis, Miss.


*Account of Diphtheritis, as it occurred on the Watershed between the Tallahatchie and Mississippi Rivers*—By Lea Z. Williamson, M. D., of Sardis, Miss.—Diphtheritis occurred in the vicinity of Sardis, Miss., in 1859, and as this affection is now attracting much attention, I will endeavor to present an account of the symptoms which it presented, and the treatment which I found most beneficial, with such other information as may tend to throw light on the subject.

*Symptoms*,—Preliminary symptoms usually precede the attack in adults, sufficiently definite to apprise an intelligent person of his danger. These were a dull aching of the bones, lassitude, headache, great mental depression, and drowsiness. Children are emphatically the subjects of diphtheritis, and these initiatory signs are rarely observed in them. More commonly the child awakens in the morning, complaining of sore throat and stiffness of the cervical muscles; he seems very sleepy, insists on being let alone, and lies with his hands folded under his head. He has some fever, little or no appetite, and inspection reveals redness of one or both fauces, and sometimes of the uvula, and tumefaction of one or both tonsils. Perhaps the membrane has already formed on some of these parts, or does so in a few hours, sometimes it does not form until the third day. Externally there is swelling of the submaxillary and cervical glands, and the degree of this is a fair and correct exponant of the internal injury. Of fifty-eight cases, the left side was first effected in forty-four; in eleven only one side was implicated. The exudation commences in small, irregularly, whitish or ash colored patches, sometimes confined to a part of the fauces, or scattered here and there over there whole extent. If these patches coalesce, the whole mucous surface is concealed by the false membrane.

Occasionally the exudation appears first on the uvula. When the surrounding surface is of a deep red, and the membrane of whitish color, the fever is sthenic; when the surface is a dark livid or claret, and the membrane of a yellowish color, the fever is typhoid; and when the latter condition succeeds the first, the prognosis is unfavorable. Between these two conditions, however, it must be remembered there are various grades wherein the characteristic symptoms are more or less mingled, and modified. We have not observed that constitution has any determining effect as regards the character of the fever.—In the majority of cases the fever was asthenic: whilst in some of the very worst cases, as regards the throat there was scarcely any fever perceptible. The membrane begins to be removed soon after it is completed, either in strips, or by softening and mixing with the fluids of the mouth. They are sometimes removed, and renewed several times; each time becoming thinner and whiter, and finally disappearing. The process lasts from five to ten days; the longer, the more unfavorable the prognosis; few recover that go to the tenth day. If it continue this long the fetid sanies from the nostrils, and the lacinating pain along the Eustachian tubes, when fluids are swallowed, indicate the extension of the inflammation into these passages, and there are reasonable apprehensions of the invasion of the larynx and trachea, which is the chief danger of the disease, and which will almost certainly prove fatal.

When the disease has advanced this far, the front of the neck, the parotid glands and the face are greatly swollen; the mouth cannot be opened without the most excruciating pain; the voice, although the tongue is not involved, is changed into a hoarse whisper; the swallowing of fluids, even, is torturing. The pulse is feeble and fluttering; the respiration is hurried and catching; the indentation of the intercostal muscles from atmospheric pressure shows a lack of oxygen in the lungs, for the relief of which the diaphram and pectoral muscles are brought into full play. The patient is restless, tossing from side to side; implores the assistance of the bystanders, or a release from the agonies of suffocation; finally seeks a semi-recling position, and dies by apnœa. The obstinate constipation so often present in the beginning, is exchanged for diarrahœa in the latter stages—the stools having a very offensive but not a cadaverous odor. Hæmaturia, difficult micturition, and suppression ot urine are also common at this time. Albuminuria is detected by the usual tests in the severe cases; but cannot be considered a constant complication. In a few cases the serous effusion of the areolar tissue of the face, neck, and chest was so great as nearly to conceal the eye, and entirely incapacitate the patient from weraring his own clothes. One case had a diphtheritic membrane, formed on an excoriated surface of left arm, which was very tenacious, and as tardy of being removed by the same remedies as that of the throat; which could not have come from a merely local affection.

The system is evidently under some poisonous influence, which has probably much to do with the fever of diphtheritis.

The prognosis was favorable in ordinary constitutions, if early treated. All the severe cases that were not treated till after the 2d or third day, died. All died that had a descent of the membrane into the larynx, with one exception. Of 58 cases, one was over 40, 5 over 30, 9 past 20, 16 over 14 years; the other 42 had not reached the age of puberty; 40 were under 10, and 20 of these between the ages of 4 and 6 years; the youngest was only 19 months old; no membrane formed in this case, though there was much inflammation and swelling. One third more females than males suffered. Color confers no immunity. 4 whites and three blacks died; one 30; two 8; one 6; two, 5; and one 4 years old. One died on the 18th, one on the tenth, one on the 9th, one on the eighth, one on the 7th, one on the sixth, and one on the third day.

*Etiology.*—Diphtheritis appeared here on a high, level water shed, between the Tallahatchie and Mississippi rivers. On each side are broad, uncultivated valleys, of matchless fertility, where grows vegetation of the richest and rankest character, which by the overflow going off in May or June, is left exposed and reeking in the sun. Superadded to this are numerous lakes, marshes, and sloughs, which are supposed to make this region notorious for intermittent and remiitent fevers. This year (1859) the summer fever commenced early in June, and prevailed about as usual until August. The season was very dry, there having been no general rain since early in May. The thermometer ranged from 86° to 96° Fahr. In the last week of July there were copious rains, with frequent showers throughout August and September. With the rains came a decided change in the temperature, the thermometer ranging from 69° to 82°. The first cases occurred August 5; a week later half a dozen families were attacked almost simultaneously, without having had any communication with the first cases. It continued to travel in a definite direction along the eastern border of these table lands, confined to very narrow limits, from which it never once deviated. Remittent bilious fever, the only disease from which the inhabitants suffer during the summer months, and which had been prevailing to its usual extent, seemed now merged into the prevailing epidemic; after the appearauce of diphtheritis, not one case of fever was seen in the epidemic region, where scores are wont to occur. A few weeks later, diphtheritis appeared on the western border of this ridge (diphtheritis never reached its centre) fronting the Mississippi bottom, differing in no respect from that already described. On this basis it is a fair inference that in this epidemic malaria and diphtheritis were in some way connected. The epidemic began to abate in September; there were fewer attacks and those of a milder form. No rain fell after September 22 until Nov. 17. The weather was uniform, and warm for the season; this had a salutory effect. Patients were always worse during wet "spells," or when nights and mornings were very cool. Frequently those that had recovered, relapsed from exposure to cold, damp atmosphere. Diphtheritis being a disease heretofore unheard of in this section, the people were terrified with stories of contagion, for which there was barely the remotest evidence.

*Diagnosis.*—Diphtheritis has been confounded with scarlet fever, black tongue, mumps, croup, ulcerated and malignant sore throat. It wants the excavated surface of ulceration of the last named disease; when, however, the false membrane has been removed and renewed several times, some excavation will be observed.

*Treatment.*—I commenced this generally with purgatives to relieve the constipation. Where there is much fever, a hot surface and clay colored stools, good results usually follow the administration of calomel in broken doses, followed by a saline cathartic. Most cases thus treated exhibit some improvement on the second or third day. Mercury is preferred as a stimulant to the secretions, and as an antiplastic to the blood. Emetics are only useful for expelling the false membrane from the larynx in the last stages, thereby preventing suffocation. Iodide of potassium was given as an antiplastic, also the chlorate of potash for the same end, and to correct the fetor.—Huxham's tinc. bark and mur. tinc. iron were beneficial in the low and lingering cases. Chlorinated soda, a drachm to three ounces of water, is an excellant gargle. The application of nitrate of Silver, solid or in solution (a drachm to the ounce), to the inflamed surface, once or twice a day, was a prominent and indispensable part of the treatment in the severe cases. Externally, the most active counter-irritants are the best applications. The merits of flies, mustard poultices, stimulating lotions, and rubefacient liniments were thoroughly tested—the same arguments urged against blistering in other throat affections apply in this. Mustard vindicates itself from these, and is decidedly a superior application; and when added to Indian meal or wheat bran poultice, can be tempered to the patient's tolerance. It alleviates the internal pain, and controls to some extent the diphtheritic exudation.

*Sequelæ.*—In several cases serious secondary affections came on after the throat had recovered, characterized by universal paleness of the skin, lips, tongue, and mucous surface, and extreme whiteness of the conjunctiva. The muscles are soft and flabby; the patient is feeble; has a sort of random shuffling gait; cannot grasp and retain bodies by the hand. There is great mental depression, and disposition to sleep; constant constipation, feeble appetite, and digestion. Neuralgic pains of neck, shoulders, and body are common. In one case sight was so much impaired that large print could not be read, and the voice was nearly destroyed. The soft palate and uvula dangled in the pharynx like a dead curtain. The larynx of this person had been severely affected. All these cases recovered under rational treatment.---*Am. Jour. Med. Seiences.*

